# Thinking, Walking, Talking: Integratory Motor and Cognitive Brain Function

**DOI:** 10.3389/fpubh.2016.00094

**Published:** 2016-05-25

**Authors:** Gerry Leisman, Ahmed A. Moustafa, Tal Shafir

**Affiliations:** ^1^The National Institute for Brain and Rehabilitation Sciences, Nazareth, Israel; ^2^Facultad Manuel Fajardo, Universidad de Ciencias Médicas de la Habana, Havana, Cuba; ^3^School of Social Sciences and Psychology, Marcs Institute for Brain and Behaviour, University of Western Sydney, Sydney, NSW, Australia; ^4^Faculty of Social Welfare and Health Sciences, Graduate School of Creative Arts Therapies, University of Haifa, Haifa, Israel

**Keywords:** motor processes, cognitive processes, cognitive–motor interaction, executive function, prefrontal cortex, cerebellum, basal ganglia, premotor cortex

## Abstract

In this article, we argue that motor and cognitive processes are functionally related and most likely share a similar evolutionary history. This is supported by clinical and neural data showing that some brain regions integrate both motor and cognitive functions. In addition, we also argue that cognitive processes coincide with complex motor output. Further, we also review data that support the converse notion that motor processes can contribute to cognitive function, as found by many rehabilitation and aerobic exercise training programs. Support is provided for motor and cognitive processes possessing dynamic bidirectional influences on each other.

## Introduction

The association between motor function and cognition can be understood, in part, in the context of the evolution of human bipedalism. Bipedalism served as a significant basis for the evolution of the human neocortex as it is among the most complex and sophisticated of all movements. It is characteristically human (even though birds on the ground, some mammals, and primates possess that function as well); thus, humans are dedicated to this mode of locomotion. Birds have a larger encephalization index than do their reptile cousins, with that difference being explainable, in part, by bipedalism (1). Bipedalism in humans is both constant and employs an upright spine, unlike other organisms with that skill. On this basis, we can conclude that the development of the large brain of humans was associated with bipedalism’s development.

We surmise that the humans have a unique ability to harness gravitational forces as a direct result of the existence of the upright position. The basis of the continuance of this genetic mutation is based on the notion that bipedalism had created larger pools of neurons. It is argued that the same evolutionary process has allowed us to develop the binding of the motor system into synchronous, rhythmic, purposeful movement, which expanded to eventually allow for cognitive binding and consciousness.

Postural muscles, we claim, were the main conduit for this motor and cognitive binding to evolve and continue to exist [for a more comprehensive review of the nature of evolutionary brain development, posture, brain size, and the implications for limitations of the pelvis as well as the genetic implications, the reader is referred to Ref. (2, 3) as well as Ref. (4)]. Reduced postural activity in childhood harms natural exploration of the surrounding, thereby reducing the ability to learn from experiences, and leading to developmental delays. Thus, deviations from normal postural development or from normal levels of postural activity can disrupt or delay cerebellar and cortical maturation and may disrupt the underlying oscillatory timing mechanisms on which motor and cognitive binding is based (5–9). As a result, cognition, more likely, evolved secondarily and in parallel to the evolution of human upright bipedalism.

Although viewed as separate functions historically, it can be argued that complex motricity and cognition are functionally connected, and both evolved in parallel, interdependently. According to Llinás (10), oscillations of neural activities can represent both motor and cognitive processes, suggesting that both processes may share similar evolutionary roots. This is supported by a recent review on shared evolution of motor and cognitive processes (11) as well as by data and analysis by Vallortigara et al. (12), arguing that evolution of attention serves successful motor processes. Along these lines, Carruthers (13) argue that working memory has developed to serve motor control in animals. It has been argued that episodic memory evolved from place cells in the hippocampus (14). Similarly, it has been argued that the basal ganglia have similar anatomical corticostriatal loops that serve both motor and cognitive processes (15). Cognitive processes became more sophisticated associated with the need for the adaptation of more complex movements (4, 8, 16–18), which probably relates to improvement in fine motor movements.

Both cognitive and motor function are controlled by brain areas such as frontal lobes, cerebellum, and basal ganglia that collectively interact to exert governance and control over executive function and intentionality of movements that require anticipation and the prediction of movement of others. Developmental disorders and other disorders of brain integration all involve disruption of executive processes, functions attributable to the frontal lobes, and articulation with motor components of the nervous system (4, 8, 19). A common symptom of developmental disabilities, for example, includes clumsiness or motor incoordination, especially as it relates to gait and posture and with strong evidence supporting the concept of “weak central coherence” or a processing bias for featural and local information, and relative failure to extract gist or “see the big picture” in everyday life [for a fuller description, the reader is referred to Ref. (20)].

Impulse control disorders, both inhibitive and facilitative, as well as disorders of executive function and judgment, either inhibited or facilitated, and judgment disorders can all be attributed to dysfunction of this network and its control of motor and non-motor cognitive behavior. In the following sections, we will discuss examples for the interactions between cognitive and motor functions.

## Cognitive–Motor Interactions: Thinking about Moving

### Embodiment

Whether one moves or one is planning to move or thinking about someone else moving, overlapping neural networks are activated. Motor–cognitive interactions involve the planning and production of action, a direct consequence of the stored memories of information necessary to anticipate and interpret the behaviors of others. Problem solving has been demonstrated to rely on these cognitive–motor interactions (8, 16, 21).

One may never have thought about how one plans and controls movement, but we know that actions, such as we might see when playing the violin or throwing a ball, writing, or eating with a knife and fork, are not simply reflexes. Movement is not simply triggered by an external stimulus such as what one does upon touching a hot stove. Movement can also be the result of a series of mental processes. These mental processes can be used cognitively even when no movement results.

Movement and action are oftentimes assumed to be the same thing with a continuity existing between planning and enactment. Movement can be defined as body parts displacement in physical space, be it voluntary or involuntary, where an action can be defined as consisting of movements necessary for goal-directed activity.

Actions are planned with respect to a specific goal. For example, if you are thirsty and want to take a sip of coffee, you might look at your coffee mug, reach toward it, wrap your fingers around the handle, lift the mug, and bring it to your lips. These motor actions implicitly involve various integrated cognitive functions that allow successful motor performance.

The findings about embodiment, which were developed from computational [e.g., Ref. (22)], neuroscience (23, 24) and behavioral (25, 26) perspectives on cognition suggest that knowledge may have evolved from perception, memory, attention, and acting (27, 28). All views of embodiment share the understanding that cognition is a complex set of internal activities, bound to each other and to the world through perception and action in real time with no static and isolated representation of anything, that is, that cognition is just a complex dynamic system.

### Motor Imagery

Further support for the connection between movement and thought involves motor imagery. Motor imagery can alter the neuronal action in the primary sensorimotor areas in a comparable fashion to that observed with an actual performed movement, where, for example, tetraplegic patients are able to operate an EEG-based control of a hand orthotic with nearly 100% classification accuracy by mental imagination of specific motor commands (29, 30).

Most bodily systems possess a dynamic interaction through feedback between different brain regions that involve feedback connections. In applying this notion to cognitive–motor interaction, one should be able to effect motor performance by cognitive imagery and cognitive performance by motor and movement exercise. Motor control and the attendant motor–cognitive processes can be readily evaluated through the use of motor imagery. Mental imagery theory indicates that cognitive–motor processes such as motor imagery and the observation of action share the same representations as motor execution (31). Munzert et al. (32) overviewed motor imagery studies that support and extend predictions from mental simulation theory. They noted that the brain’s motor regions are significantly involved in motor imagery or in the cognitive representation of movement and action. Mental training procedures, for example, can be readily applied as a therapeutic tool in motor function rehabilitation (33).

It has been demonstrated in other electrophysiological studies (34) that brain activity during motor imagery is comparable to activation with the performance of actual movement. Sitaram et al. (35) had observed that the primary motor cortex was active in similar ways with both actual and imagined movements, and Filimon et al. (36) reported similar results in relation to the dorsal premotor and superior parietal regions, as well as the intra-parietal sulcus.

The general idea is that motor imagery is part of a wider notion of the “motor representation” linked to the intention and preparation for movement. The normally unconscious process of motor representation can be conscious under some circumstances. A motor image is a conscious motor representation. By this characterization, motor images possess the same characteristics as those of the motor representation, that is, they have the same functional relationship to the imagined or represented movement and the same role in the creation of this movement [a more fully developed view of this process is reported in Ref. (37)].

If one were to ask whether other aspects of the movement–thought connection are relevant to any discussion of mental imagery, one of necessity must include an understanding of the physiological correspondence between actual motor activity and intention, preparation, and execution of motor acts and the mental imagery equivalent. For some time now, there has been strong evidence for the physiological equivalence of imagery and motor acts. Electromyographic activity (EMG) has been found to significantly increase during motor simulation. Jacobson had long ago (38) found micro-movements and increased EMG in those limbs involved in imagined movements, but not in the contralateral limbs. A long time ago, Shaw (39) also reported proportional EMG increases to the extent of imagined exertion, supporting the assertion that the kinesthetic mental image of a motion configuration is accompanied by the same innervation pattern found in the motion itself (40, 41).

Mental recreation of movement triggers motor output (31, 37). Bonnet and colleagues (42) had subjects either press isometrically on a pedal, or mentally simulate the same action. Weak and strong levels were used with the main result of this experiment being that spinal monosynaptic reflex excitability was increased during mental simulation at approximately the same level as for the actual movement. Also, the change of reflexes in the limb imagined to be involved in the movement was larger for a strong than for a weak simulated pressure.

Decety et al. (43), Decety and Grezes (44) examined normal subjects visualizing a graphic movement (writing “one, two, three,” etc.). The subjects were instructed to imagine the movement from the “first person perspective” and to try to “feel their writing hand.” Brain areas corresponding to the prefrontal cortex, supplementary motor areas (SMA), and also the cerebellum were activated significantly, as well as the basal ganglia. PET data obtained by Fox et al. (45) and Gerardin et al. (46) demonstrated that imagined finger movements stimulate the SMA and parietal areas bilaterally. Actual movement activated the contralateral sensorimotor cortex as well as the SMA and parietal areas on both sides. Stephan et al. (47) also noticed that during mental imagery, the SMA foci were located more anteriorly than during execution. Confirmatory data were also obtained by the functional Magnetic Resonance Imaging (fMRI). Sanes (48) and Sanes and Donoghue (49) studied fMRI activation during both executed and imagined finger movements. They found that, whereas anterior motor areas (including the SMA) are activated in both conditions, M1 is activated only during execution. Hallett et al. (50), also using fMRI, reported that activation occurred in primary motor cortex during imagined movements. The level of activation was less during imagination than during execution. Finally, Decety et al. (51) reinvestigated this problem using PET and summarized those findings (44) in 2006. Three-dimensional graspable objects were presented to subjects who were told to visualize clutching the objects with their right hand. A significant increase in regional cerebral blood flow (rCBF) was noted in areas concerned with motor behavior. At the cortical level, area 6 in the inferior frontal gyrus was significantly activated on both sides. Activity also significantly increased in the left prefrontal areas that also included the dorsolateral frontal cortex (areas 9 and 46), and in the parietal lobule (area 40). In addition, the anterior cingulate cortex (areas 24 and 32) was activated bilaterally. At the subcortical level, the caudate nucleus significantly activated bilaterally as was the cerebellum but only on the left.

### Motor Cognition

Motor cognition (i.e., cognitive processes that underlie complex motor output) encompasses the mental processes involved in the planning, preparation, and production of our own actions, as well as the cognitive processes involved in anticipating, predicting, and interpreting the actions of others. Motor–cognitive interactions can be best understood through the perception–action cycle involving the transformation of perceived patterns of intended movement into coordinated patterns of actual movement. For example, toddlers who do not perform this function well, or some of those who have suffered a stroke impairing their ability to descend a flight of stairs with automaticity, will descend one step at a time. We, on the other hand, descend one step and “know” that the remaining steps each possess the same riser-height as the preceding step. We casually notice and compute how high each step in a stairway rises, and accordingly, we lower our feet assuming the riser height to be the same for each subsequent step (52).

Even the seemingly simple movement planning required for descending a staircase – unconsciously determining when and to what degree to lower one’s foot – relies on a sophisticated set of neural processes. In terms of evolution, the function of perception is not simply sensory interpretation and recognition of objects and events, but we are additionally provided with guidance and feedback for our movements, allowing for the efficiency and optimization of that movement for goal-directed behavior to be achieved appropriately and efficiently.

In addition, the planning of movement is not a unidirectional process from perception to action, but rather involves feedback from our movement that aids us in planning and executing subsequent motor action. It is not just that perception exists partly in the service of planning movements; our movements allow us to perceive change, which in turn allows us to plan our subsequent actions or movements. Accordingly, the relationship between motor and perceptual processes is bidirectional. This is how normally functioning adults and not toddlers or some poststroke patients possess automaticity in descending a staircase.

Shared coding and functional connections between these processes in the nervous system accomplish the integration of the control of perception and action. Actions and movements necessary to achieve a goal require cognitive plans which, in turn, have both perceptual and motor components (8, 9, 53).

## Why Specialization of Cognitive and Motor Brain Regions?

### Cognitive–Motor Processing

**Figure 1:**
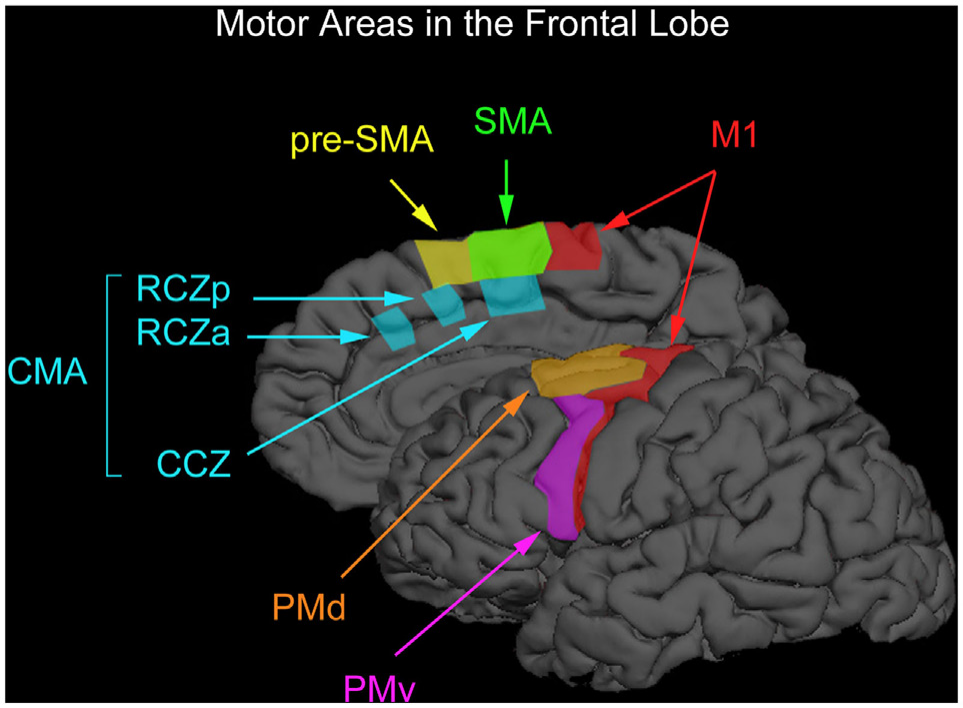
**Motor areas in the frontal lobe**. The premotor cortex consists of the ventral premotor (PMv) and dorsal (PMd) regions. The medially located supplementary motor and presupplementary motor cortex are designated as preSMA and SMA. The regions below the superior frontal sulcus are where the premotor cortex can be found (PMv) and that part of the premotor-labeled PMd cortex located above the PMv. The boundary between the SMA and preSMA is the vertical anterior-commissural line [after Chouinard and Paus (54)].

Motor cognition is localized in brain areas responsible for movement control. Motor processes in the brain are supported by various control centers, with the principal area consisting of M1, which is the “lowest level” motor area for the control of fine motor movements, and with fibers directly innervating the muscles themselves. In addition, the premotor area (PM) is associated with the linkage of specific motor program sequences (with input sent to M1), and the SMA in order to support the creation and execution of action plans. One can, therefore, view these processes in a hierarchical fashion with M1 at the lowermost point and the SMA at the highest. We can consider these regions and their articulations, on the one hand, from aiding in the processing of elemental and simple sorts of information, such as the specific movements associated with M1, to more complex and precisely defined sets of movements associated with the PM, to eventually, for supporting plans for articulated and complex movements necessary for goal-directed action in the SMA. These three areas are illustrated in Figure 1.

The neural activity associated with motor response preparation in the M1, PM, and SMA has been examined to observe the differences that exist between tasks initiated by external (turning off an alarm clock) vs. internal stimuli (setting an alarm clock). Internally generated stimuli require an advanced strategy to execute a movement, whereas externally generated stimuli require no advanced planning in order to execute a movement. Mushiake et al. (55) recorded single-cell activity from the M1, PM, and SMA of monkeys, immediately prior to and during the execution of sequential motor activity. Movement sequences were either triggered internally (IT) or visually (VT). The monkeys were required to touch three buttons on a panel as they were lit in a random order, during the VT condition. With IT stimuli, the monkeys were required to recall the predetermined order and press that sequence on a keypad without visual feedback.

In both IT and VT conditions, M1 neurons showed no significantly different activity during both pre- and movement conditions. One can logically conclude that the results were obtained due to the fact that both conditions required the production of the same movements. However, when comparing the IT to the VT condition during both pre- and movement periods, more neurons were active in the SMA in the IT when compared with the VT. These findings have been supported in more recent work in humans (56–58).

It seems then that the SMA allows for the formulation of motor planning. The PM seems to be responsible for organizing specific movement sequences, as PM neurons are significantly more active in VT as compared to IT during premovement as well as in movement periods. We can conclude, therefore, that motor production and behavior occurs at multiple processing levels, and one can observe differences in neural processing when one formulates a plan in advance and when one simply responds to an environmental cue. The discovery that these three brain areas operate on increasingly more specific information might suggest that the areas always operate strictly in sequence; specifically, it might be tempting to think that SMA finishes processing and only then directs the PM, which finishes its processing and only then in turn directs M1. But this apparently is not the case. Instead, other neural evidence suggests that the three brain areas do not always operate in this sequence, but instead interact in complex ways.

Nevertheless, different brain regions play different roles in the conception, initiation, and control of action. We have already seen that the SMA is involved in the organization of motor sequences based on plans, and that PM is involved in the preparation of a specific action. In addition, the prefrontal cortex and basal ganglia are involved in the initiation and in the temporal organization of action, and the cerebellum is involved in the temporal control of action sequences [cf. Ref. (8, 19, 59)]. The SMA, PM, and M1 regions are active when movement anticipation is required. Actually, motor feedback networks from “sending” to “receiving” regions allow for the mirroring of activity that allows for the integration of diverse brain regions to act coherently.

Cognitive–motor interaction then, is part of a multicomponent system, with simultaneously occurring discrete processes associated with various but specific brain regions in the normal individual (9, 60).

### Functional Networks of the Brain in Cognitive–Motor Interaction

We have seen how motor and cognitive functions interact to support purposeful movement. Specific brain areas are less important anatomically than the nature of interregional communication and networking within the brain and nervous system. Cognitive–motor function is allowed through the interaction of disparately located brain regions in the adult brain, with hemispheric specialization being the developmental result of the necessity of the adult brain to optimize motor, cognitive, and perceptual skills.

Early childhood is marked by a lack of localized brain function and over the lifespan human skills become controlled by regional centers as a way of effecting better and optimized cognitive and motor performance.

Childhood brain development is characterized by exuberant connectivities between brain regions that continue throughout early development and even through adolescence [cf. Ref. (61, 62)]. Neurological development in childhood allows for relatively rapid learning that will eventually be optimized for how well brain regions function in tandem, based on the effectiveness of neuronal connectivities. Current research has demonstrated that functional connectivities relate directly to cognitive functions associated with memory and reading ability [cf. Ref. (4)].

Infancy is characterized by clumsy and non-optimized motor behavior and similarly by less integrated cognitive performance. Localization of function in the brain is the result of a need for automated and optimized performance in the adult requiring efficient local rather than multi-focal control (4, 9, 63–67). It is the function of the brain maturation process to facilitate optimized and integrated adult cognitive and motor function.

“Functional specialization” is a notion that indicates that a given region of the cerebral cortex is dedicated to, in the case of our discussion, the control of specific cognitive or motor function thus allowing for both its functional and anatomic separation from the surrounding cortical areas.

For example, Brodmann’s area 4 is defined by its borders that include the medial longitudinal fissure medially, the precentral sulcus anteriorly, the central sulcus posteriorly, and the lateral sulcus laterally. It was Penfield and Boldrey (68) in 1937 who described the specialized function of this region as resembling a homunculus, in which the trunk and legs double over the midline; the hand and the arms are represented in the middle, and the face at the base. Brodmann labeled this area as a separate functional entity due to its discrete cytoarchitecture (69). Otfried Foerster was one of the first scientists to note that within this area “stimulation of a given focus produces a single isolated movement of the corresponding part of the body” [(70), p. 137]. Since then, an overwhelming number of studies have used cortical stimulation approaches or functional neuroimaging techniques, and investigated in great detail the functional properties of that area, which was later termed the “primary motor cortex” (M1) (71–74).

However, localizing activity in a distinct cortical region does not explain how spatially distributed areas are bound together for mediating and/or sustaining a cognitive or motor process. Functional specialization is therefore only meaningful in the context of “functional integration” (75, 76). The concept of functional integration assumes that sensory, motor, or cognitive processes rely on context-dependent interactions between different brain regions based on, according to Friston (77), precise anatomical and functional connectivities. For example, M1 activity may be triggered by either facilitatory or inhibitory premotor stimuli that in turn interact with stimulation from sensory, posterior-parietal, or prefrontal regions (78–80).

As there is so much competing information impinging on the system at any given moment, there exists a requirement to reduce potential interference while competing tasks or information is being processed. One way that effective optimization of information processing can be accomplished is by the functional separation of brain areas both within and between functional systems (81).

In addition, there exist numerous theories of brain organization that all support and describe brain function as a consequence of network function. These theories include Kinsbourne (82) conceptualization of brain organization as being a consequence of the inhibition of competitive feedback and interhemispheric rivalry. Kinsbourne’s conceptualization has been examined through neural network, theoretical, and experimental models. Reggia et al. (83) in their model suggested that subcortical competitive processes may be a more important factor in cerebral specialization than is generally recognized. They indicated that there is a dispute in the wider experimental literature about whether trans-callosal interhemispheric influences in the human brain are primarily excitatory or inhibitory. Some experimental data are apparently better described by assuming inhibitory callosal influences. Past neural network models endeavoring to study this matter have faced the same dilemma: in intact models, inhibitory callosal influences best explain strong cerebral lateralization like that occurring with language, but in lesioned models, excitatory callosal influences best explain experimentally observed hemispheric activation patterns following brain damage.

The more complex nervous systems evolved out of simple nerve nets. Giant neurons radiate giant fibers, which quickly and wholly communicate to the caudal musculature, to program, among other things, swift escape movements in reaction to danger. Where a response is particular and discriminating, the anatomy of its output mechanism reflects that fact. An example is the direct projection of corticospinal fibers onto motor neurons that control digital movements in humans and those species in which these movements are differential but not when they are not, such as in cats. Another example is the absence of direct trans-callosal association between Betz cells representing the fingers in humans, but their presence in other mammals (84, 85).

Kinsbourne’s argument essentially involves the effects of spreading activation by central inhibition, a concept supported by the analysis of Koch and Leisman (86). This mechanism can be modified, according to Kinsbourne, by experience with the developing brain inhibiting preprogrammed fixed action patterns (FAPs) (87) and mass responses. Contrary to the suppositions of stimulus–response and cell assembly theories, the child’s brain is not a *tabala rasa* expecting imprinting by its owner’s life experiences. Instead, neural connections are “prewired” in a genetically determined species-specific fashion. In its ability to orientate, acquire knowledge, approach and withdraw, the infant is highly limited in a lawful way. The infant turns toward weak and away from strong stimuli (88). Approach, withdrawal, and startle all involve stereotyped interactions that use many muscles in an invariant pattern of relative contraction and relaxation or FAPs. The infant’s powers of perceptual discrimination and its response selection are inadequate to provide for an independent existence. The developing infant becomes competent not by any expansion of the brain.

Behavioral diversity features the growing ability of the infant to deviate from predetermined patterns of input processing and output, and to represent and mentally manipulate information and action that are increasingly independent of overt postural change. Selective inhibition is critical in these respects. The ability to deviate from “prewired” highly probable responses is based on inhibiting them, so that less probable response can successfully compete for control of behavior.

The ability to detach attention from salient stimuli (and therefore to both attend to subtleties and organize one’s perceptual search in a logical and systematic, rather than stimulus-bound fashion) depends on the ability to inhibit (or habituate) one’s attention to what is salient. Similarly, the ability to move differentially, without triggering all of the interactions in which the movement is rooted, is contingent upon inhibiting the unnecessary components of the synergism. To stop a single movement, rather than let it continue, becomes achievable only in association with the maturation of the motor system.

It has been thought for sometime, as Goldstein (89) commented that, “Movements continued to (their) extreme are simpler than those which must be stopped at a certain point.” It is the inhibitory component that lets brain maturation contribute to behavior. The ability to entertain novel hypotheses presupposes the ability to inhibit the neuronal pattern basic to more obvious, but ineffectual, solutions. The ability to recollect a previous experience is contingent on the capability to detach attention from the more noticeable impressions of the present. The same applies to the ability to solve problems by mentally representing possible solutions. The ability to plan and that includes movement, is contingent upon being able to hold concurrently in mind distinct items of information, making it possible to combine them inventively in various ways until their relationship assumes a configuration that approximates the intended objective. This integration of information into a plan can only be accomplished if the items of information held their specificity for purposes of their integration. They can only retain their specificity if each underlying neuronal representation is protected from distorting crosstalk. Thinking then connects with movement.

### Optimizing Integratory Functional Network Organization in the Brain

Numerous authors conceptualized and provided evidence to support the notion that oscillatory patterns propagate and coordinate cross-neuronal interactions (90–93). What emerges then is a clear consensus that network theory is a useful means for describing and elucidating brain function. Therefore, it is more appropriate to apply a connectivity-based systems approach to explain the neurobiology of brain function in normal, pathological, and developmental states as opposed to localization or specialization models and methodologies that have anatomically segregated regions controlling specific behaviors. Further, functional deficits in cognitive–motor function are more likely the result from problems in the functional networks rather than dysfunction in a localized area, the former resulting in disorders of optimization and efficiency rather than in a complete loss of function. We are, therefore, capable of examining improvements in optimized function as well. This we will examine later as a consequence of the effects of movement and exercise on brain function and cognitive performance.

In an attempt to understand the nature of integratory brain function, we can start by observing the nature of integratory function in the cognitive skill of language from an integratory rather than localizationist perspective. Although the left hemisphere is nominally dedicated to the control of the language function in most individuals, patients with damage to the right hemisphere have comprehension difficulty with linguistic units with multiple meanings, and with the understanding of connected discourse, they fail to use broad contextual cues or information (94).

We know from event-related potential studies in neurologically intact individuals that semantic processing triggers right-hemispheric activation, whereas syntactic processing primarily activates the left hemisphere (95). Patients who have had their left hemispheres surgically excised due to significant pathologies demonstrate that their right hemispheres can support numerous language functions, although it is only the left that can support normal syntax. When taken together, these findings imply that the right hemisphere in adults is involved in pragmatics and semantics, the left hemisphere alone is the province of the control of syntax.

There are three major distinguishable components of syntax that relate to motor function: (1) the principal categories of words (nouns and verbs, with the dependent categories of adjectives and adverbs); (2) ordering of words, including sub-ordering, that is, the clustering of words within a larger order; and (3) function words (including sub-words e.g., morphemes such as terminations of abstract nouns, verb inflections, etc.). The syntax of a language results from the co-operation and interaction of these three components. A motor theory of language has motor programs and the principles for combining motor programs as the underlying structure of language. There also exists a close link between motor control (action organization) and perception (the organization of vision). For each of the three components in syntax, the relation to motor theory may take the form of: (1) a relation directly with the organization of action (referred to by one writer as “the grammar of action”); (2) a relation directly with the organization of perception (referred to by Gregory years ago as “the grammar of vision”) [(96), p. 622]. Vision, of course, is motor-based with discontinuities created by the combination of movement of fixation, saccades as well as a constant tremor all together playing an important function in maintaining the stability of visual processing (97).

In children, language acquisition is more importantly represented in the right hemisphere as compared to language processing. If prior to language acquisition the child suffers brain damage, right hemisphere insult is more damaging to future language acquisition than damage to the left hemisphere (98, 99).

Another difficulty with concluding simply that the left hemisphere is the language organ is that although the left hemisphere is primarily responsible for language, in adults it is not specifically dedicated to it. Actually, there have been suggestions that the left hemisphere function is specialized for controlling the performance of well-practiced routines [cf. Ref. (100)].

**Figure 2:**
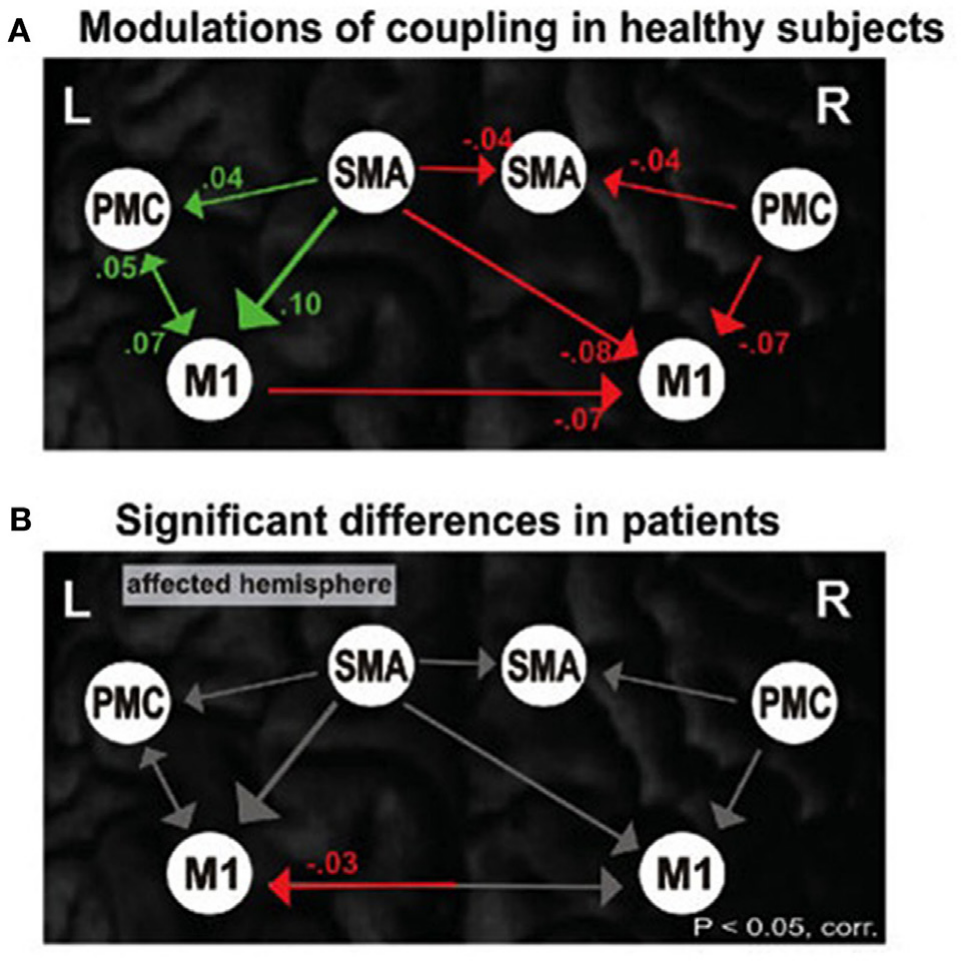
**Motor network connections during unimanual hand movements in (A) healthy individuals and (B) significant differences in functional coupling during affected (right) hand movement in individuals with stroke**.

The fewer brain regions necessary for the control of an operation, the more optimized or efficient the function, but that is not to say that there is no further control possible if those areas are destroyed or dysfunctional. If that were the case, then there would be no basis for rehabilitation and recovery after stroke for example (8, 65). Specialization is necessitated by the brain’s need to optimize control. Integratory function within the system is still an issue of coupling as represented in Figure 2 where we can see effective connectivity of motor networks during unimanual hand movements [cf. Ref. (101)] compared with inefficiencies associated with individuals poststroke.

## The Association of Cognition and Movement

One of the primary functions of neurological development of the nervous system is the integration of developing systems so that function will be localized for more efficiency. But that is not to say that the system must work by localized control (102, 103). For example, the languages that are learned in early childhood prior to the development of Broca’s and Wernicke’s areas, with their nominal control of expressive and receptive language respectively, are learned fast as a consequence of the exuberant neuronal connectivities present in early childhood development (8, 65).

These abundant connectivities in childhood allow for the rapid acquisition of knowledge. The system of exuberant connectivities in childhood, as a result, renders the nervous system of the child less optimized than the adult brain state and its resultant localization of function. When that now optimized localization of function has developed by adulthood, the number of potential connectivities is significantly reduced. Specialization of cortical regions optimizes the system but does so by concentrating the networks in a circumscribed area allowing for more effective temporal as well as spatially represented responses. In short, more potential connectivities in early childhood will lead to greater automatization of skill-development and localized function in the normal adult and less of an ability of the adult to acquire information with as much ease as in early childhood.

The concept of “cortical efficiency” that has been described elsewhere (66, 67, 104, 105) indicates that more efficient neural processing is associated with greater skill in performing cognitive tasks, but that increased ability is not necessarily associated with the activity of a given brain region involved in that processing. One might expect greater activity to be associated with better performance. However, as it relates to the function of the cerebral cortex, the opposite has been found. Enhanced performance in verbal (106), numerical, spatial, and figural reasoning (107, 108) is associated with reduced energy utilization in various cortical regions.

In electrophysiological studies, examining coherence in various frequency bands, background power in the 7.5–12.5 Hz band, for example, diminishes with cognitive tasks compared with resting state. This decrease has also been noted in subjects with higher IQs as compared to those with lower, as well as with individuals who have been tested prior to practicing a particular cognitive or motor skill over a period of time and retested after the skill’s acquisition. Such findings would indicate a more efficient processing strategy for the performance of cognitive tasks (60, 62, 109, 110).

Child development facilitates the creation of functional specialization in adulthood, the principal purpose of which is to facilitate optimized cognitive and motor functioning. The ability to dynamically alter these abilities renders them as a result plastic. Movement facilitates brain plasticity and the development of interregional associational networks and therefore influences cognitive–motor interaction (111, 112). The localization of function is not the explanation of how cognitive processes are controlled in the brain, but rather represent the end-result of practice. Directly related to the efficiency of cognitive function is the effectiveness of network function and organization in the cerebral cortex, which is now measurable (8, 9). Network efficiency is comparable across individuals as a direct result of the number of brain regions necessary to accomplish a single task. The fewer brain regions necessary to accomplish a single task in one individual renders that individual more efficient or optimized, perhaps in some sense even more intelligent for that task compared to another who requires greater use of more expansive networks as well as the increased latency from stimulus onset to response.

Both cognitive and motor functions require the learning of sequential actions. These sequences are most optimized with control by specialized networks mediated by both executive function and automaticity (113, 114). The learning of complex sequences requires adequately functioning executive processes (e.g., those involved with error monitoring or motor program structuring). Structural complexity remains the same for any sequence. Activations at varying levels of complexity have demonstrated overlap in the supplementary motor cortex (115) and other brain regions, such as the cerebellum, basal ganglia premotor cortex, thalamus, ventrolateral premotor cortex, and precuneus, with increased activations at increased levels of complexity (8).

**Figure 3:**
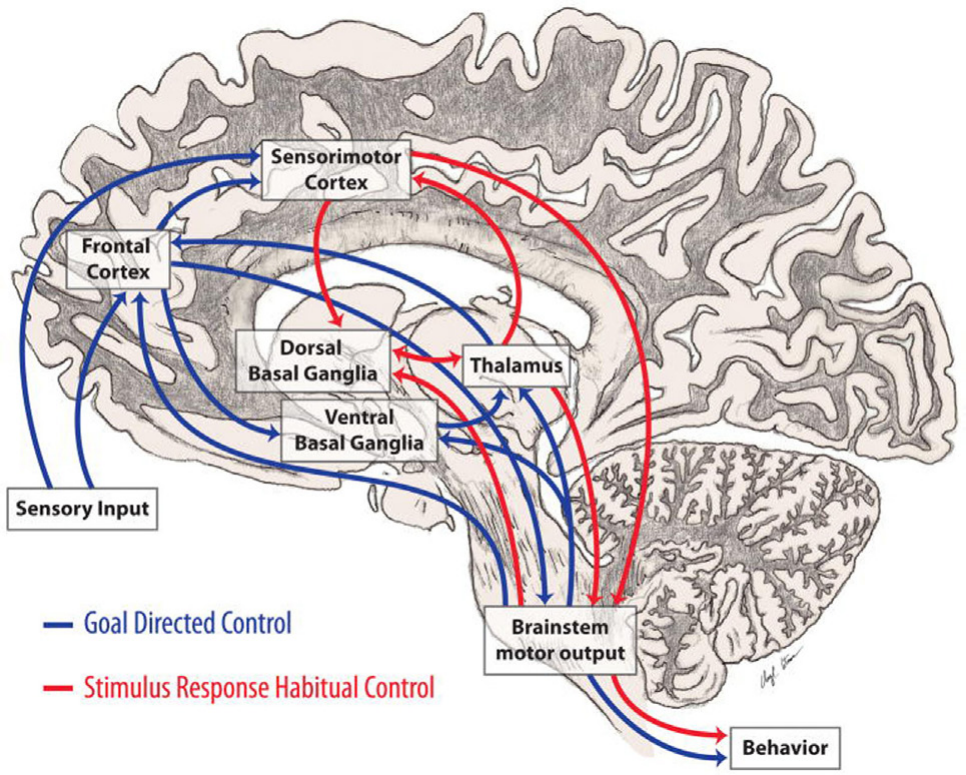
**Automatic and voluntary (cognitively interacting) motor control**. Motor control integrates both cortical and subcortical structures principally involving those connections between the basal ganglia and frontal lobes involved in automaticity of motor function and its cognitive mediation. (8). In Parkinson’s disease, loss of dopamine in the caudal basal ganglia leads to impaired automatic movements involving circuits important in stimulus based habitual learning (red arrows) and over-reliance on cognitive components of motor control and circuits involved in reward based learning (blue arrows) (from http://neuroanatomyblog.tumblr.com).

Executive function and action intersect and co-operate with each other (8). Useful actions are initially acquired during childhood and youth, and continue to be acquired throughout the course of life, by means of incidental experience and by formal education (110). There is obvious advantage to automate actions such as walking down a flight of stairs as compared to, playing a tennis match, or the violin. Voluntary (i.e., cognitively interacting) vs. automated action are described in Figure 3.

**Figure 4:**
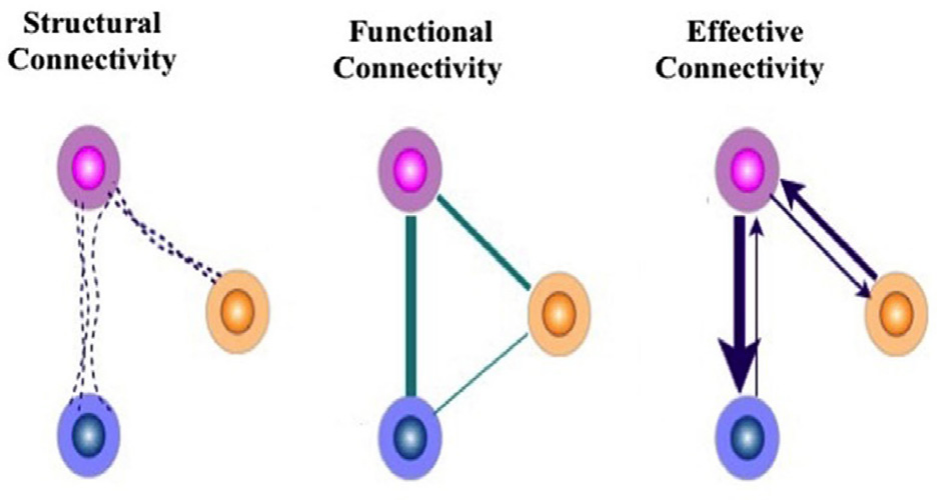
**Modes of connectivities described based either on FMRI or qEEG measurements as a basis for evaluating efficiencies of connections**. The figure on the left exemplifies the fiber pathway structural connectivity, the functional connectivity (correlations), and effective connectivity (information flow) between brain regions during analysis of electrophysiological or functional imaging data. From these connectivities, we may be able to find the roles of natural phenomena as brain connectivity refers to patterns of either anatomical links, statistical dependencies, or of casual interactions among distinct areas within a nervous system. The study of the human connectome allows us to better understand the pattern of anatomical links, statistical dependencies, and/or casual interactions among distinct areas within a nervous system.

To understand better how connectivity analysis can allow us to examine processing efficiencies in cognitive motor interaction, we must pin down the concept of brain connectivities (116). Brain connectivity can be divided into three main concepts: (a) anatomical (or structural) connectivity measured in terms of physical (and chemical) connections between neuronal populations or individual neurons, (b) “functional” connectivity by which we mean the statistical similarity between activities in distributed neuronal populations, and (c) “effective” connectivity, according to Sporns (117), addresses the effect that a given brain region exercises on another region in organizing coherent responses. The distinctions described above are useful in that the measurements and analytical methods allow us to examine each component separately (118). Effective connectivity can be defined as the effect one node of neurons exercises on another (119). We can derive effective connectivity by employing a model of neuronal integration by approximating model parameters that best explain observed EEG/MEG or fMRI signals. Functional integration of behavior then can be effectively evaluated by measurements of effective connectivity by the dynamic examination of the model of neuronal coupling. It is exemplified in Figure 4.

**Figure 5:**
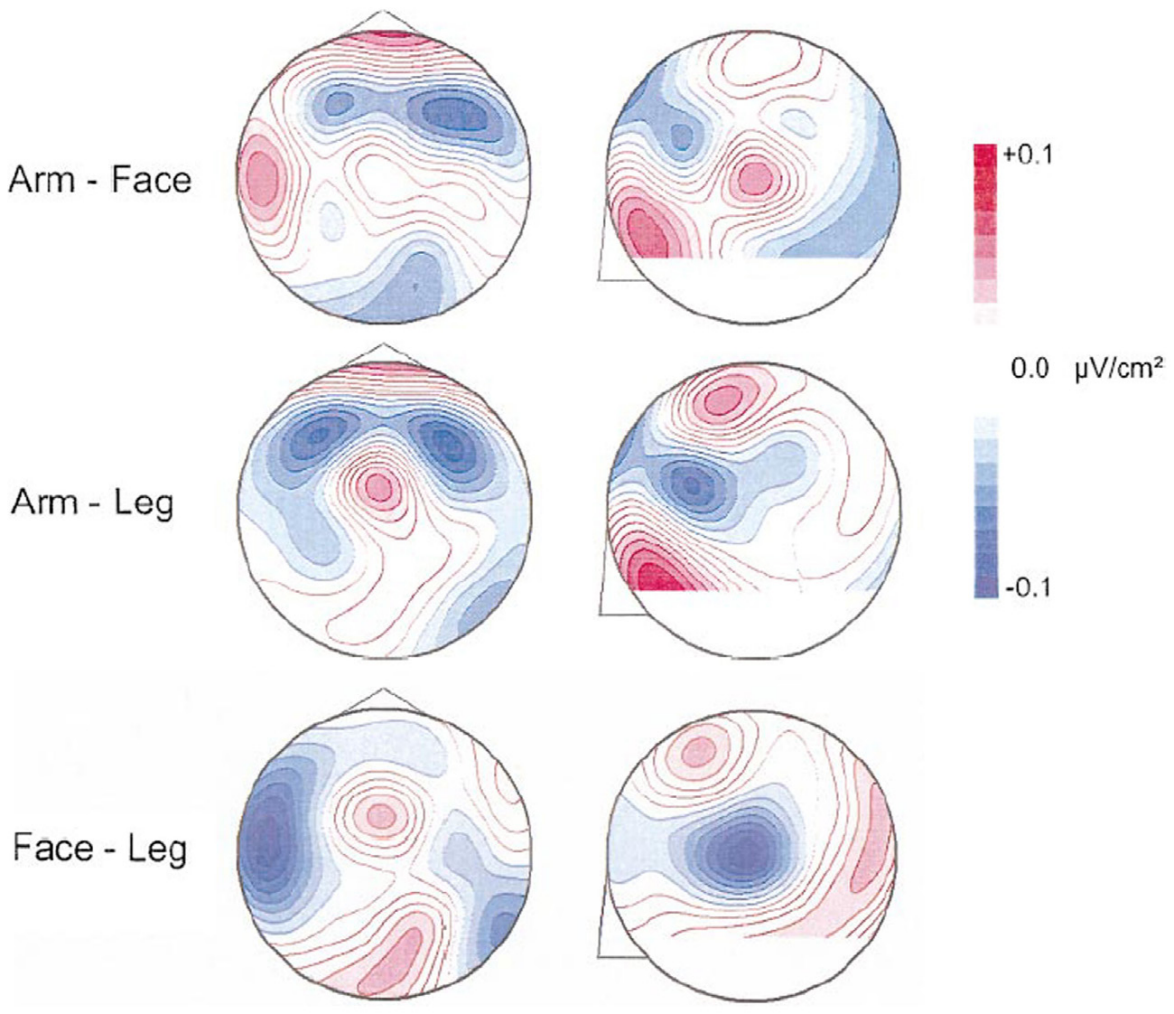
**Presentation of difference maps of potential source densities (PSD) for 240–260 ms**. Data for face- and leg-related words were subtracted from arm-related words. The circles on the left represent the head from above (nose = up; left = left). Circles on the right characterize lateral views on the left half of the head (nose on left). Red foci indicate stronger ingoing potentials for face-related verbs (upper diagrams) and leg-related verbs (lower diagrams). Blue foci indicate stronger ingoing activity for arm-related words. PSD is enhanced at left-lateral locations for face words and at central sites for leg words. At 240–260 ms, direct comparison of CSD topographies is provided elicited by face- and leg-related verbs. The view from the top is shown on the left and a lateral view on the left hemisphere is presented on the right. Stronger ingoing potentials for face (leg) words are specified in blue (red). Greater ingoing potentials are present at left-lateral sites for face-related items and at central sites for leg-related items [cf. Ref. (126)].

Because of the linkage between motor and cognitive function that we represent here, it is our contention that inactivity has an effect of rendering an individual’s cognitive as well as motor performance less efficient or utilizing significantly decreased modes of functional and effective connectivities and exercise has the converse effect (8, 120–125). Figure 5 demonstrates significant motor system activation with different action verbs using qEEG (126) and supporting the application of the relationship between language function with action visualized or actualized. Pulvermüller et al. (127) recorded brain electrical activity evoked by visually presented words using dipole current source density localization. Verbs referring to actions usually performed with different body parts were compared. Significant topographical differences in brain activity elicited by verb types were found starting at 250 ms after word onset. At the vertex, close to the cortical representation of the leg, leg-related verbs (for example, to walk) produced strongest ingoing currents, whereas for face-related verbs (for example, to talk) the most ingoing activity was seen at more lateral electrodes placed over the left Sylvian fissure, close to the representation of the articulators. Action words caused differential activation along the motor strip, with strongest in-going activity occurring close to the cortical representation of the body parts primarily used for carrying out the actions to which the verbs refer.

**Figure 6:**
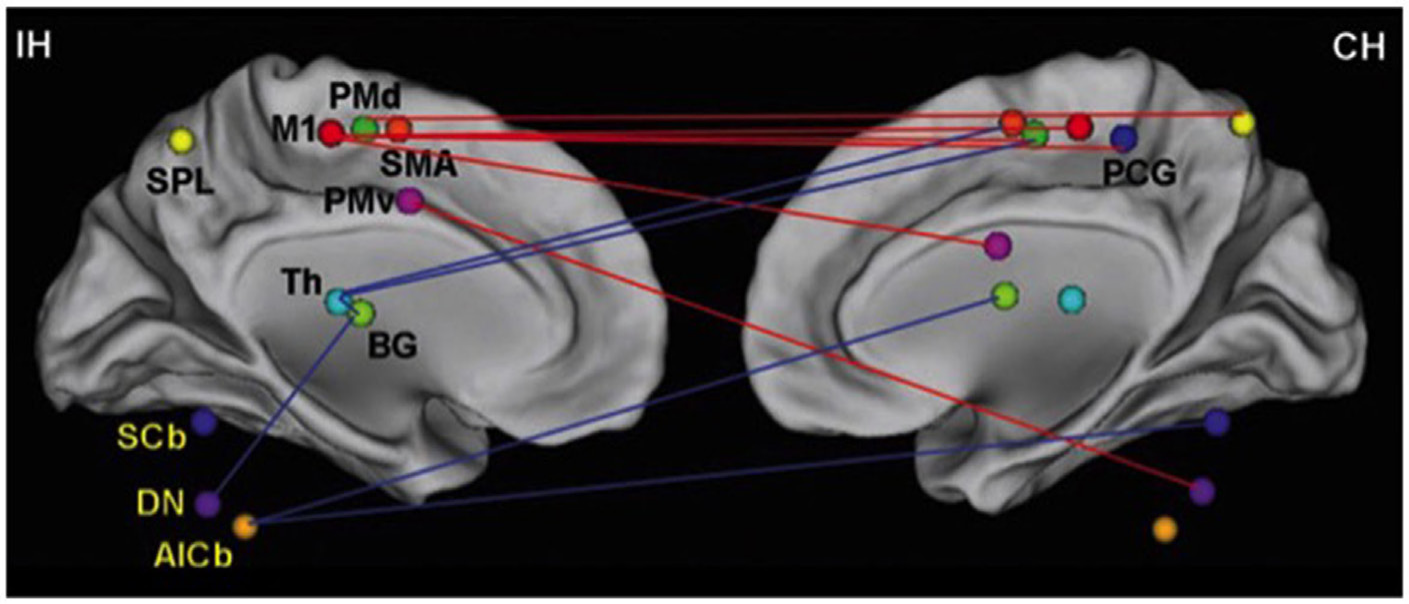
**Changes in network efficiency over time post-stroke**. Connectivity parameters between nodes of the motor network show increased connectivity (red lines), which are primarily seen as interhemispheric connections between M1 and contralesional sensorimotor regions. Reduced connectivity (blue lines) is mainly found in ipsilesional subcortical areas and cerebellum. IH, ipsilesional hemisphere; CH, contralesional hemisphere; M1, primary motor cortex; PCG, post-central gyrus; PMd, dorsolateral premotor cortex; PMv, ventrolateral premotor cortex; SMA, supplementary motor area; Th, thalamus; BG, basal ganglia; SPL, superior parietal lobule; SCb, superior cerebellum; DN, dentate nucleus; AICb, anterior inferior cerebellum. Reprinted with permission from Wang et al. (128).

What trauma or disease can do to cognitive–motor interaction is to render the networks between the two sets of skills less efficient in ways similar to the inefficiencies seen in early child. The concept of “rehabilitation” speaks to that notion with habilitating for a second time. Figures 2 and 6 demonstrate connectivity parameters between nodes of a motor network that changes the efficiency of the network as a consequence of stroke.

## Effects of Physical Activity on Cognitive Performance

We had seen above that cognitive exercises and motor imagery can effect overall motor performance (129, 130), but does the reverse hold true? Can motor training and exercise affect cognitive performance? The “mind” and its attendant cognitive abilities is no longer simply conceived of as a control mechanism for logical/abstract functions, but rather as a biological system interconnecting bodily experience and action and how those functions allow interaction with other individuals. From this perspective, the physical–mental dichotomy cannot be simply understood in the context of action and representation, but must be seen as closely interrelated, perhaps even part of the same process. Action, the interaction with objects, and the co-operation with individuals in our world; the representation of the world as well as perceiving what is in it, categorizing it, and understanding the significance of perceptions, are different levels of the same relational link that exist between organisms and the local surroundings in which they operate, live, and think. This is reflected both developmentally: in the effects of motor development on cognitive development and throughout life.

Developmentally, it has been shown that children significantly late in achieving developmental motor markers are at high risk for later cognitive impairment (131, 132). In a large British general population birth cohort, Murray et al. (132) examined available data of infants, children, and adolescents motor and language function, as well as general intellectual abilities and neuropsychological performance that included executive function and verbal fluency. Murray and colleagues observed that more rapid accomplishment of motor developmental milestones was highly associated with increased cognitive performance in adulthood, especially in the area of executive function.

The developing infant develops a sense of self and of independence by exploring his surroundings and navigating to objects of curiosity and interest. The principle question concerns the influence on a proceeding (or currently planned) muscular act (133). That influence arises from motivation-generated expectation of the act’s consequence, and it is thought to succeed only if “consciousness” is present (134).

Prior to the accomplishment of the balanced upright position by the child and his first steps, there would have been numerous unsuccessful attempts, with the inevitable attendant pain. The discomfort that the child perceived would be stored as a memory that would subsequently modify certain self-paced movements. With repeated drill and self-initiated attempts, the child will arrive upon the precise combination of basic and essential movements and timing allowing him to take his first steps. Deposited temporarily into explicit memory is the consolidation of complex patterns of motor activity (135), and later transmitted (136) to long-term implicit memory, likely during the many recurring periods of sleep (60, 109), characteristic of infancy. Rapidly, the toddler is capable of walking without having to concentrate on every step, as we do when descending a staircase.

Movement continues to affect cognition even after initial motor and cognitive development. We have reinterpreted the role of the motor system within the overall schema of the central nervous system through the recent research on canonical and mirror neurons, thereby allowing us to pass the mind–body dichotomy of thought and action. Internal simulation of motor acts during imagery or observation of others’ movements enable social cognition, empathy, and understanding of others’ intentions and emotions (137), as well as affect one’s own emotions (138). But beyond the cognitive–motor interaction at the brain level, movement itself can affect cognition.

Garbarini and Adenzato (139) conducted a meta-analytic study to study the relationship between aerobic fitness training and the cognitive abilities of healthy but inactive older adults. The authors incorporated into their analysis the results of studies published between 1996 and 2001. They concluded that aerobic fitness activities had significantly positive effects on cognition, with the greatest benefits being observed in executive function. The results support the notion that cognitive function and neural plasticity is maintained throughout the lifespan and that a relationship exists between fitness and cognitive function.

Physical activity improves cognitive function and brain plasticity (140). The significance of this relationship is even more important than ever given the increase in aging populations with declining health and cognitive functions. Kramer and Erickson (141, 142) evaluated the hypothesis that physical activity and exercise might serve to protect, and also enhance, cognitive and brain function across the adult lifespan. They critically reviewed the literature of the effects of physical activity and exercise on cognition, brain function, and brain structure of adults, including epidemiological or prospective observational studies, randomized human clinical interventions and non-human animal studies. They noted that the literature supports the claim that physical activity enhances cognitive and brain function, and protects against the development of neurodegenerative diseases. It is not apparent from the research, the amount of exercise required and the duration of the beneficial effects. McDonnell et al. (143) recently conducted a study in which the results supported the notion that a single 30-min period of brisk exercise are associated with increases in brain plasticity with demonstrable improvement in declarative memory and motor-skill coordination.

**Figure 7:**
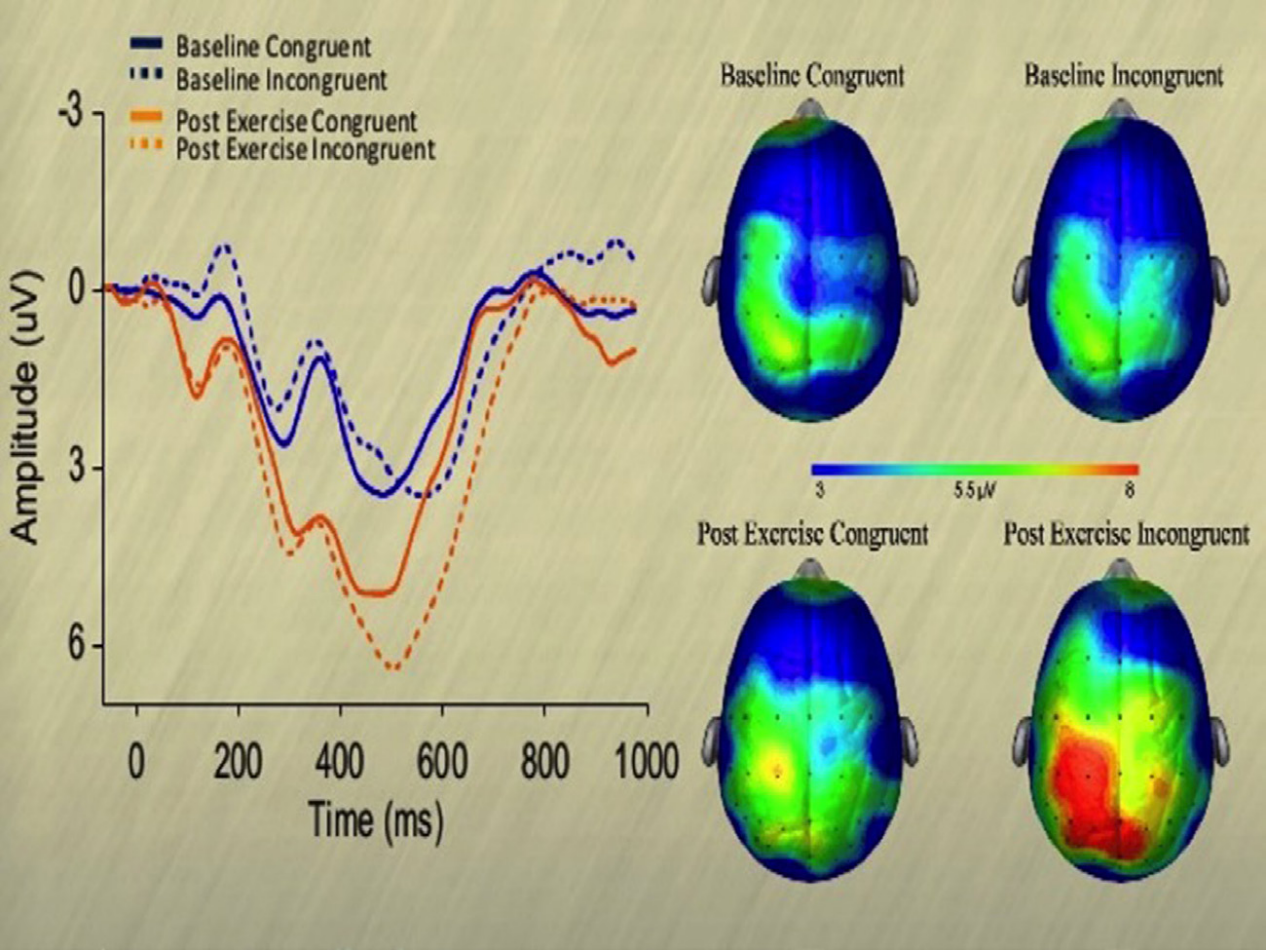
**Topographic maps of the P3 component as a function of session and task congruency on a modified Flanker task assessment of inhibitory control [cf. Ref. (145)]**. Two trials were presented, one congruent and the other incongruent that necessitated participants to press a key corresponding to a centrally presented target arrow. Congruent trials contained an array of five arrows all facing in the same direction and the incongruent trials had the four contiguous arrows facing in the converse direction to the target arrow [cf. Ref. (144)].

Hillman et al. (144) examined the relationship between electrophysiological aspects of attention and school-based academic performance and acute moderate treadmill walking. During the resting session, the investigators examined the cognitive function of the participants as well as having evaluated the cardiopulmonary fitness to determine each participant’s aerobic capability. The exercise period entailed 20 min of walking on a motor-driven treadmill at 60% of estimated maximum heart rate followed by cognitive function assessment once heart rate returned to within 10% of preexercise levels. The results showed a significant increase in response accuracy; greater P3 amplitude demonstrated in Figure 7, and significantly better performance on academic achievement tests following aerobic exercise when compared to the resting session. When taken all together, the results of this study demonstrate that aerobic exercise improves cognitive function as measured by tests of attention and academic performance. These data suggest that single bouts of exercise affect specific fundamental processes associated with cognitive function necessary for effective functioning across the lifespan.

Transcranial magnetic stimulation (TMS) is widely used to study the properties of corticospinal pathways. In recent years, it has also been used to study cortical reorganization in response to interventions such as amputation, afferent stimulation, motor learning, cortical, and spinal lesions ischemia and limb immobilization [reviewed in Ref. (146)]. Smith et al. (147) recruited a small group of adults in their late 20s and early 30s who were asked to ride exercise bikes for a period of 30 min. Changes in the brain directly after the exercise session were monitored and again 15 min later. Cortical stimulus-response curves [90–150% resting motor threshold (RMT)] were investigated as well as short-interval intra-cortical inhibition (SICI) before and at 0 and 15 min following 30 min of ergometer cycling at low-moderate or moderate-high intensity. Results demonstrated that the brain’s plasticity, or its capacity to change physically and functionally, could be improved by a single 30-min session of physical activity with noticeable changes after only 15-min of exercising. The importance of plasticity as the chief mechanism for the promotion of learning, verbal, and procedural memory, and motor behavior cannot be understated (61). Neuroplasticity underlies the actual number as well as the strength of connections between neurons as well as connectivities between brain regions. It is on this basis that we can explain why it is that exercise and physical activity positively increase cognitive–motor function.

Chaddock-Heyman et al. (148) at the University of Illinois at Urbana–Champaign found benefit from systematic exercise for the brain’s white matter and more importantly noted the facilitation of connectivities between diverse areas of gray matter in the cortex. The investigators examined the relationship between an individual’s physical fitness and brain state in 24 9- and 10-year-old children. The investigators noted thicker and denser white matter among those children who were more physically fit than others and that in turn was associated with a significantly greater facility for memory, attention span, and cognitive efficiency.

Exercise can effectively preserve brain health and cognitive function prior to the time that individuals reach the “Fourth Age.” Randomized clinical trials, for example, have shown that moderately intense exercise positively affects cognitive function with other studies showing that greater higher fitness levels and greater amounts of physical activity are associated with greater white matter integrity (149), gray matter volumes (150, 151), reduced rates of brain atrophy (147, 152), increased prefrontal cortex (153) and hippocampal volume (154), improved memory and executive function (155–157), as well as increases in brain network connectivity (65, 158–160), and lowered risk of dementia (161, 162). [For a broad review of the consequences of physical activity throughout the lifespan, cf. Ref. (163)].

The public health implications for children of cognitive motor interaction are significant. There exists a pandemic of physical inactivity among all age groups. Recent reports forecast that inactivity will continue to rise throughout the industrialized world over the next few decades (164). Although the consequences of physical inactivity on health are well known, its effects on cognitive and brain health are only beginning to emerge.

Kamijo et al. (124) investigated inhibitory control and spatial working memory in a large sample of preadolescents whose aerobic fitness was determined using the PACER test. Importantly, even though using a field test of aerobic fitness, the investigators found a significant relation between children’s cognitive skills, working memory, in particular, and fitness in general that previous investigations had uncovered using primarily laboratory measures (165, 166).

**Figure 8:**
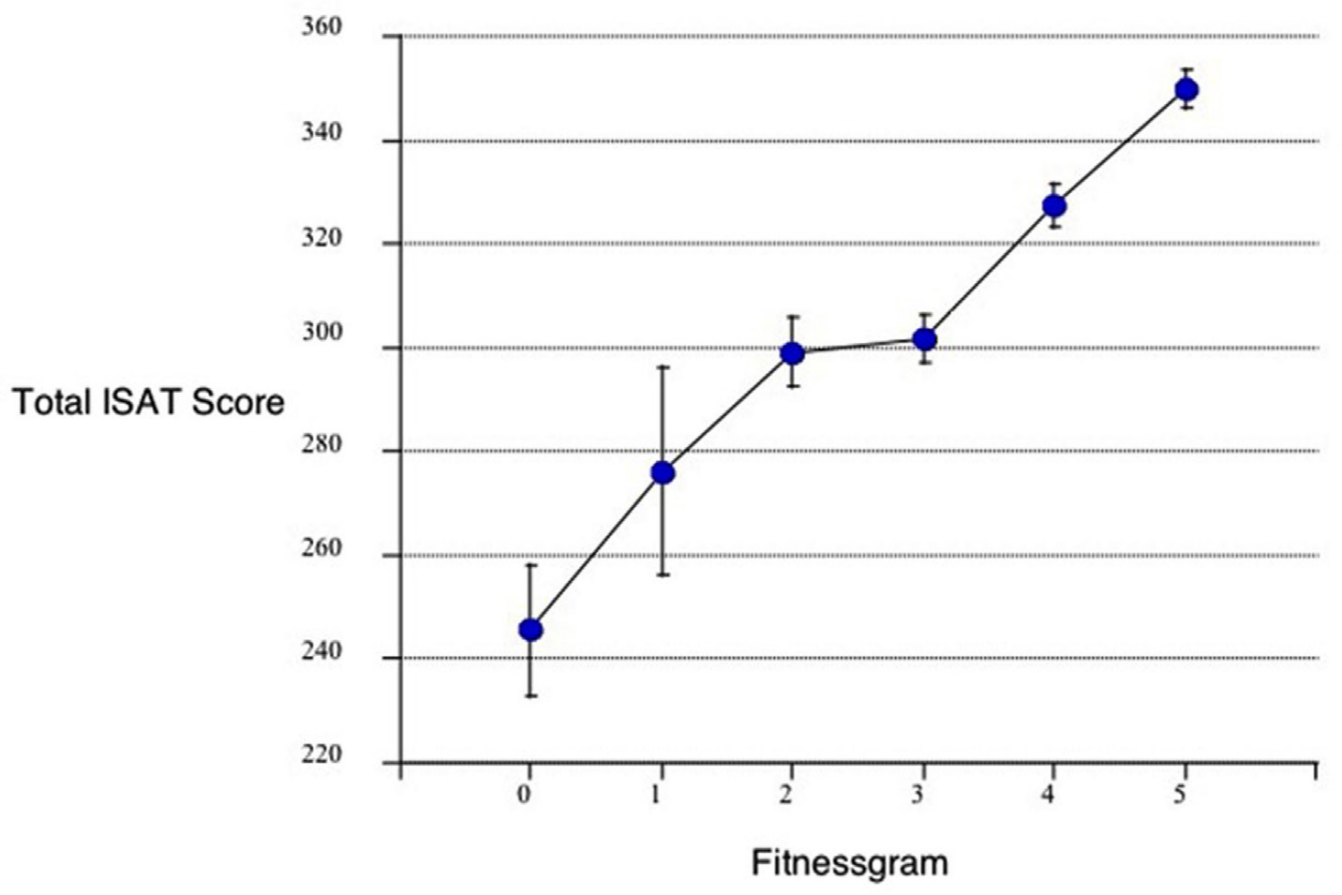
**Relationship between the total ISAT (Illinois Standards Achievement test measuring reading mathematics and science) score and the number of fitnessgram tests in which student scored in the healthy fitness zone**. The Fitnessgram is a composite of aerobic capacity (PACER), muscle (strength and flexibility), and body mass index here in 259, third to fifth school children controlled for age, sex, socioeconomic status, and physical fitness [from Castelli et al. (120)].

Castelli et al. (120), in a relatively large sample, found a relationship between physical fitness and achievement test performance in third–fifth graders. Their “Fitnessgram” was based on aerobic capacity (PACER), muscle (strength and flexibility), and the participants’ body–mass index. The results are represented in Figure 8.

**Figure 9:**
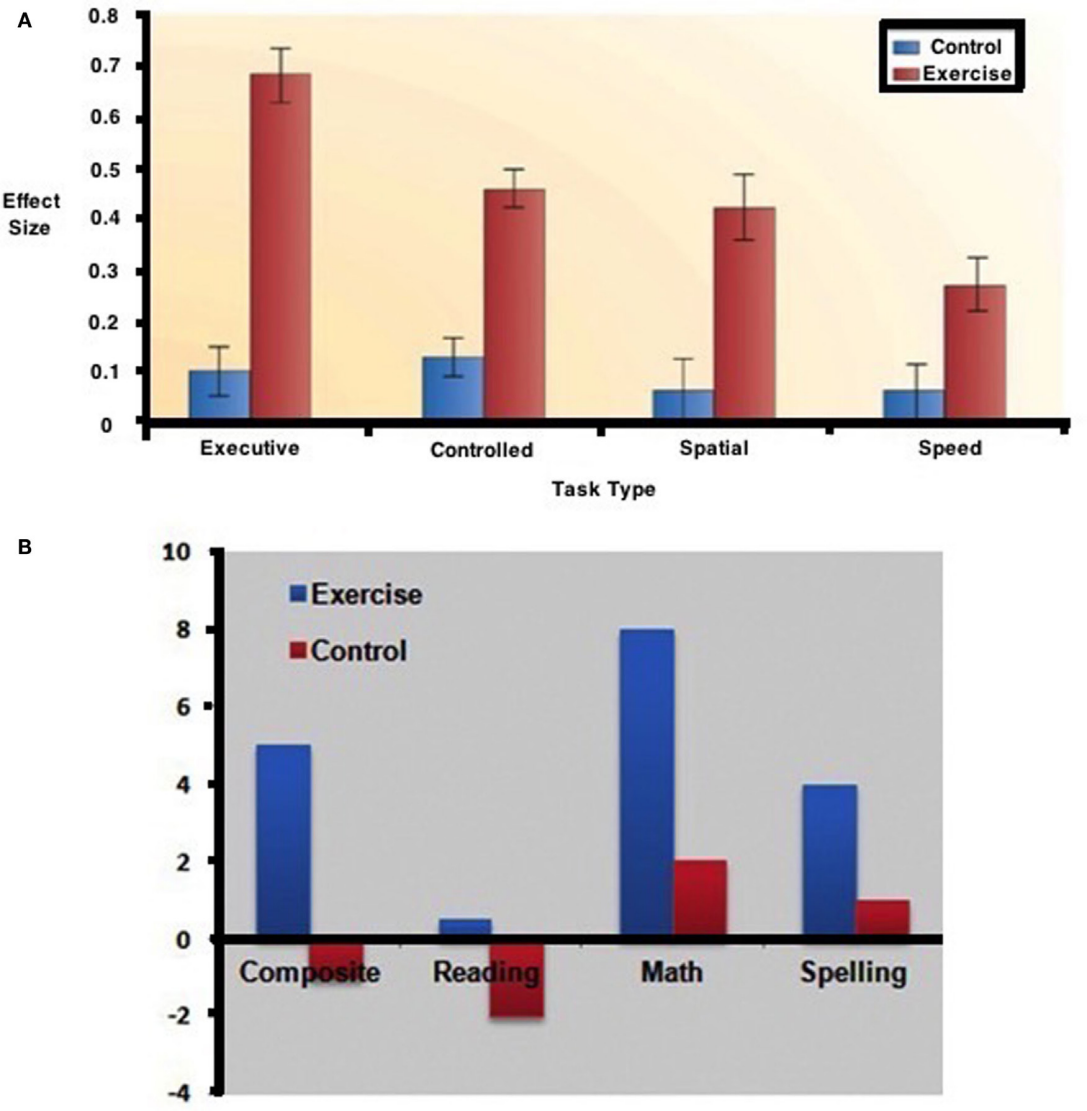
**Effects of exercise on (A) cognitive performance**. **(B)** academic skills [from Ref. (165)].

Kamijo et al. (124) had examined the relationship between physical exercise and academic achievement as did Hillman et al. (165) with a summary of the results reflected in Figures 9A,B.

**Figure 10:**
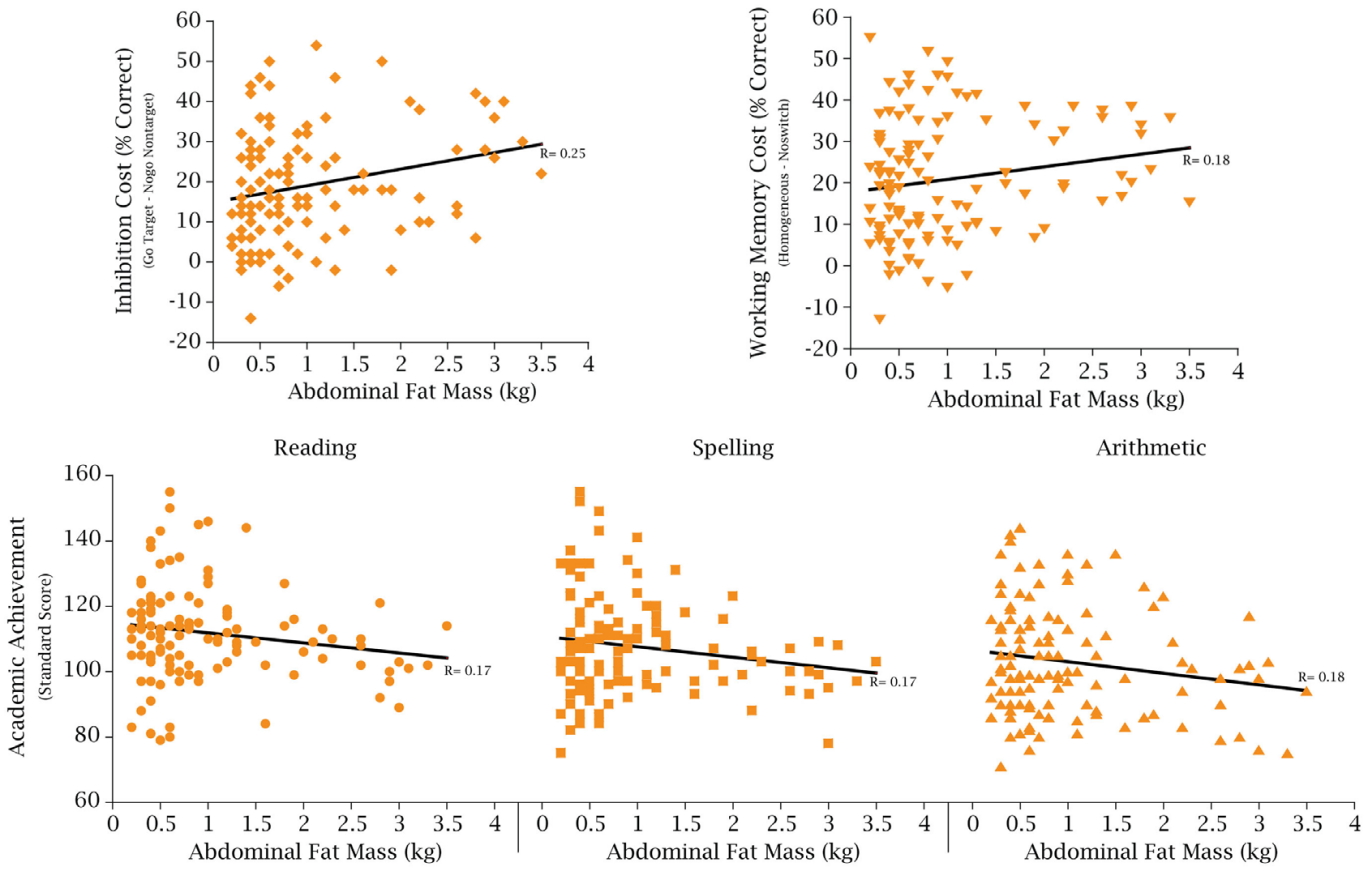
**Adiposity, cognition, and academic achievement**. The relationship between abdominal fat mass (in kilogram) on cognitive inhibitory control, working memory, and academic achievement (*N* = 122 children between the ages of 7–9 years controlled for age, sex, fitness, socioeconomic status, and IQ) [from Ref. (124)].

In further evidence of the significant effects of obesity and adiposity on academic performance, Kamijo et al. (124) noted significant relationships between adiposity, cognition, and achievement as reflected in Figure 10.

**Figure 11:**
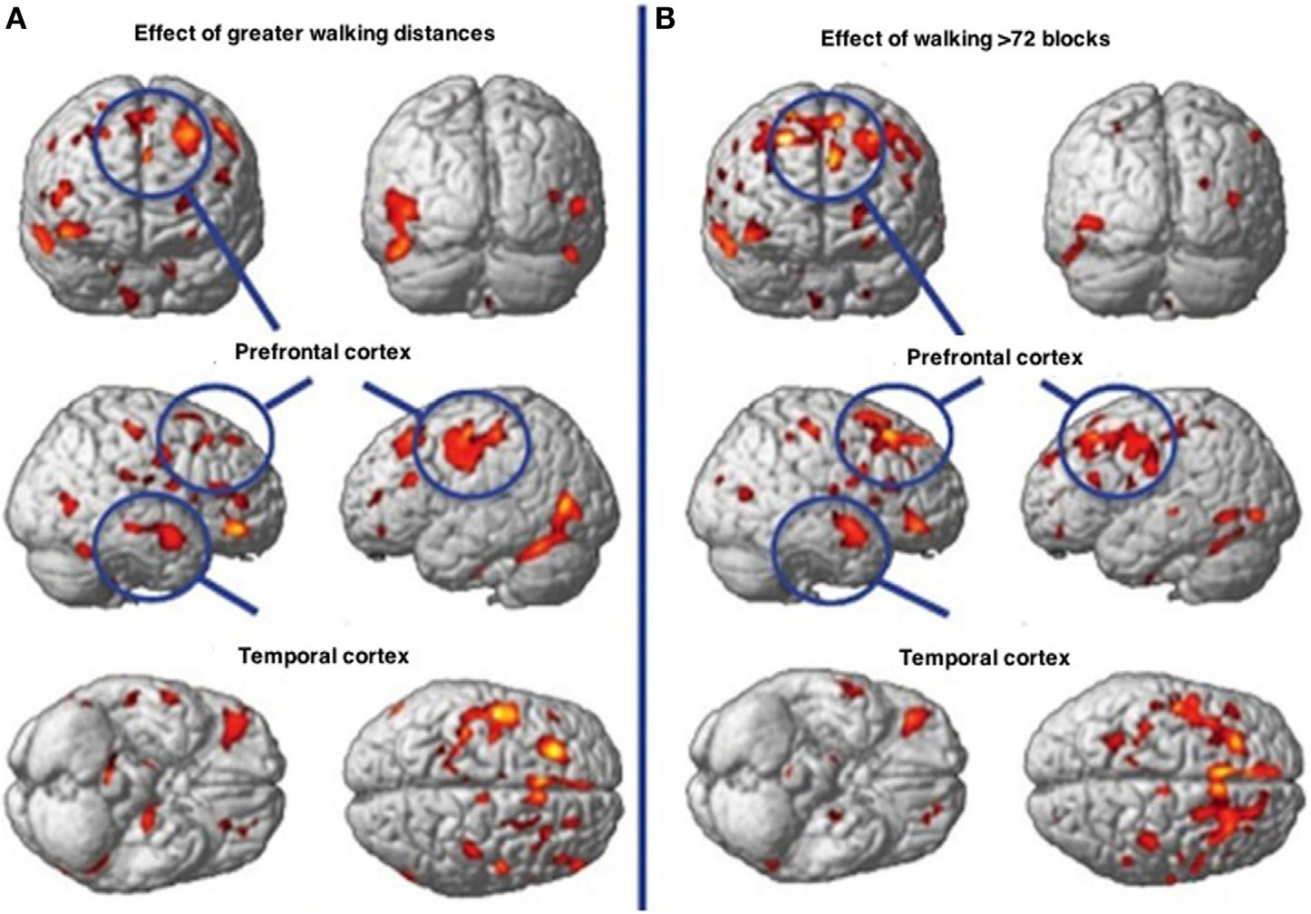
**Walking associated with gray matter volume increases in specific brain regions**. **(A)** Demonstrates the effects of physical activity on the brain at baseline. **(B)** Demonstrates the effects of walking greater than 72 streets showing greater gray matter volume.

In Figure 11, physical motor activity effects on brain at baseline are noted in contrast to the effects of walking in excess of 72 blocks with the differences being represented in greater gray matter volume. Of interest is the fact that Cotman and Berchtold (167) have found that with exercise, brain-derived neurotrophic factor (BDNF) that encourages synaptic development and differentiation in the hippocampus and basal forebrain and which is vital to learning, memory and thinking, is significantly increased in rodents. Of interest is the fact that although BDNF increases in mouse hippocampus after 7 days of volunteer wheel running compared to sedentary mice (167), discontinuation of the exercise according to (168) reverses the increased cell number as well as the cognitive gain in rodents.

## Conclusion

We have attempted here to create a basis for the characterization of cognitive–motor interaction. We reviewed data showing that the neural systems that subserve motor development also contribute to the development and operation of cognitive processes later in life. Factors related to efficiencies or optimized performance of such systems may be reflected in both rapid motor developments early in life and subsequently in improved cognitive functions throughout the life span (131, 134).

However, a number of questions remain concerning the specificity of associations between early motor development and later cognitive functions as well as between adult motor activity and cognitive function. For example, is early motor development associated with other developmental domains, such as language? Are cognitive–motor interactions necessarily restricted to executive function or does this relationship also play out in general intellectual ability? Does the association between motor and cognitive function in infancy continue into adulthood and throughout the “Fourth Age?”

As discussed above, the cognitive–motor system invokes a likely outcome of a planned motor pattern, and rejects it if the prediction is unfavorable. Before an action is made, we can perform a simulated outcome of that action, below the threshold for movement initiation that requires communication between the motor system and the muscle spindles (169). The interaction serves as the basis of sensation, always, under normal circumstances, influenced by anticipation or expectancy. “We can think without acting, act without thinking, act while thinking about that act, and act while thinking about something else” (8).

From the above reviewed data, we conclude the following: our acts can be composite, several muscular patterns being activated concurrently, though we appear not to be able to simultaneously maintain two streams of thought (170). When we think about one thing while doing something else, it is always our thoughts, which are the focus of attention. This suggests that there are at least two thresholds, the higher associated with overt movement and the lower with thought. Assuming that the signals underlying competing potential thoughts must race each other to a threshold (171), it may be significant that cortical and thalamic projections do not form strong loops (172). The presence of strong loops could make overt movement too automatic. We can now add a second possible penalty; thoughts might otherwise establish themselves by default. One should note that overt movement and mere imagery, that is, covert preparations for movement, appears to involve identical areas (173).

The bottleneck in sensory processing (174) arises because the anticipation of movement requires the avoidance of antagonism between individual muscles involved in action. As our actual and simulated muscular movements are the only means that we have to study and learn about the world, our imagined and actual movements and actions allow us to produce the unity of conscious experience. The direct result of this is that cognitive function and intelligence becomes a gage for the effectiveness of consolidating elementary overt or covert movement into more complex patterns of motor activity.

Thoughts then, according to this scheme, are simply virtual interactions that we each have with our environment, in order to create and enhance novel implicit memories and create new standard paths from sensory input to allow motor output or new optimized complex reflex patterns. Multiple routes could very well be the basis of the interaction between implicit and explicit brain function. We can conclude then that movement facilitates cognition throughout the life span.

## Author Contributions

GL, AM, and TS each contributed equally to the writing of this paper.

## Conflict of Interest Statement

The authors declare that the research was conducted in the absence of any commercial or financial relationships that could be construed as a potential conflict of interest. The reviewer CE declared a shared affiliation, though no other collaboration, with one of the authors TS to the handling Editor, who ensured that the process nevertheless met the standards of a fair and objective review.
